# A Breast Mass That Was Not Breast Cancer: Chest Wall-Invading Non-small Cell Lung Carcinoma Presenting as a Retroareolar Tumor in a Male Patient

**DOI:** 10.7759/cureus.106814

**Published:** 2026-04-10

**Authors:** Daniela Prado Escobar, Kian Memari, Claudia Pedreira, Shane Williams, Peter Cohen, Lissette P Lazo

**Affiliations:** 1 Internal Medicine, Nova Southeastern University Dr. Kiran C. Patel College of Osteopathic Medicine, Fort Lauderdale, USA; 2 Family Medicine, Palmetto General Hospital, Hialeah, USA; 3 Family Medicine, Nova Southeastern University Dr. Kiran C. Patel College of Osteopathic Medicine, Fort Lauderdale, USA

**Keywords:** breast mass in males, chest wall tumor, extramammary breast mass, non-small cell lung carcinoma, squamous cell carcinoma (scc)

## Abstract

A painful retroareolar mass in a male patient may initially suggest a primary breast process, but in rare cases it may reflect contiguous spread from an intrathoracic malignancy. Distinguishing between these possibilities is essential because delayed recognition may affect staging, resectability, and treatment planning.

We present the case of a 50-year-old man with a history of tobacco use who presented with a progressively enlarging and painful right retroareolar mass. Physical examination revealed a firm, fixed, tender subareolar lesion measuring approximately 5 cm. CT of the chest demonstrated a heterogeneous soft tissue mass involving the right anterior chest wall, with extension through the intercostal space and intrathoracic involvement. Surgical exploration revealed a tumor extending through the chest wall deep to the pectoralis major muscle, with contiguous growth into the subareolar region. Debulking and biopsy were performed. Frozen section analysis was suspicious for squamous cell carcinoma, and final histopathologic evaluation demonstrated non-small cell lung carcinoma (NSCLC) with immunophenotypic features of hepatoid differentiation. The lesion was deemed unresectable because of extensive local invasion into surrounding structures. A permanent venous access port was placed, and the patient was referred for systemic platinum-based chemotherapy with immunotherapy after multidisciplinary staging and oncology evaluation.

This case highlights a rare clinical presentation of lung malignancy manifesting as a retroareolar mass in a male patient and emphasizes the importance of maintaining a broad differential diagnosis when evaluating fixed, painful chest wall and breast-region lesions.

## Introduction

Breast-region masses in male patients are uncommon, and the differential diagnosis includes both benign and malignant etiologies. Although gynecomastia is the most common benign cause, malignant entities such as primary male breast carcinoma, lymphoma, metastatic disease, and direct extension from adjacent malignancies must also be considered, particularly when the lesion is fixed, painful, or associated with atypical imaging findings [1,2].

Direct invasion of the chest wall or breast region by a nonmammary malignancy is unusual. Lung cancer, especially non-small cell lung carcinoma (NSCLC), is well known to invade adjacent thoracic structures, including the pleura, ribs, intercostal musculature, and chest wall [3,4]. Chest wall invasion occurs in approximately 5%-8% of lung cancers and generally reflects locally advanced disease [3,4]. By contrast, breast-region involvement by lung cancer is far less common and has been reported predominantly in isolated case reports and small imaging series, underscoring the rarity of this presentation [5,6].

Because a breast-region lesion may be the most clinically apparent finding, thoracic malignancy can be overlooked when the initial focus is limited to primary breast pathology. Cross-sectional imaging and tissue diagnosis are therefore critical for clarifying the true site of origin and guiding management [5]. We present a case of chest wall-invasive NSCLC with hepatoid differentiation presenting as a painful retroareolar tumor in a male patient.

## Case presentation

A 50-year-old man presented to the ED with a progressively enlarging mass in the right breast associated with localized pain. He reported that the mass had increased in size over several months and had recently become increasingly tender. He denied nipple discharge, overlying skin ulceration, fever, or trauma. His symptoms had progressed from mild focal discomfort to persistent localized pain, with increasing awareness of chest wall fixation, prompting emergency evaluation.

His past medical history was significant for chronic hepatitis C infection. He had a 21-pack-year smoking history but had quit smoking six years before presentation. Family history was notable for lymphoma in a sibling and throat cancer in a maternal cousin. There was no documented family history of male breast cancer, lung cancer, hepatocellular carcinoma, or a known hereditary cancer syndrome.

On examination, he was hemodynamically stable and in no acute distress. Inspection of the right breast revealed a firm, tender retroareolar/subareolar mass without overlying skin changes. Palpation demonstrated a fixed lesion involving the anterior chest wall, measuring approximately 5 cm in greatest dimension clinically. No appreciable axillary lymphadenopathy was present. His Eastern Cooperative Oncology Group (ECOG) performance status at presentation was 2 because pain and reduced functional capacity from the chest wall mass limited routine physical activity but did not render him bedbound.

**Figure 1:**
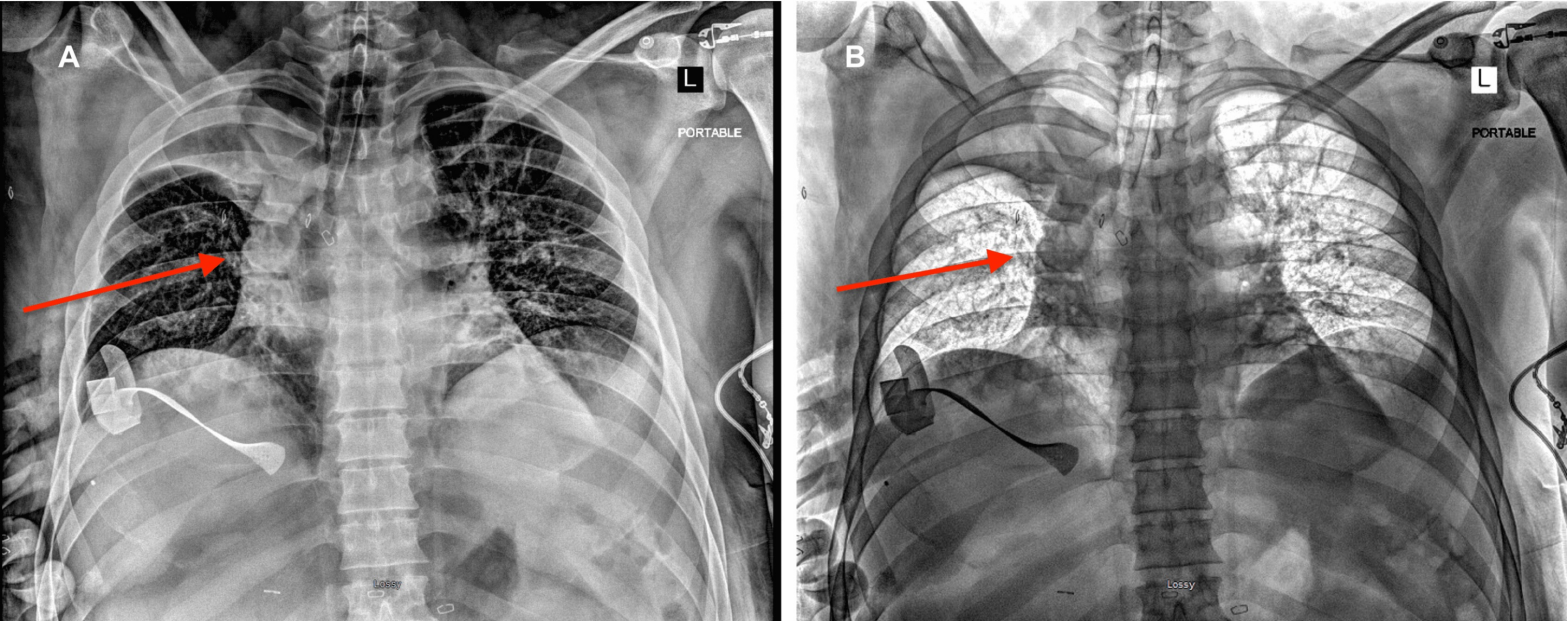
Chest radiography without inversion (A) and with inversion (B). Portable chest radiographs demonstrating an abnormal opacity involving the right upper chest wall region (red arrows), corresponding to the location of the invasive chest wall tumor seen on CT imaging. Figure 1B is shown in inverted contrast to better delineate the invasive chest wall tumor.

**Figure 2:**
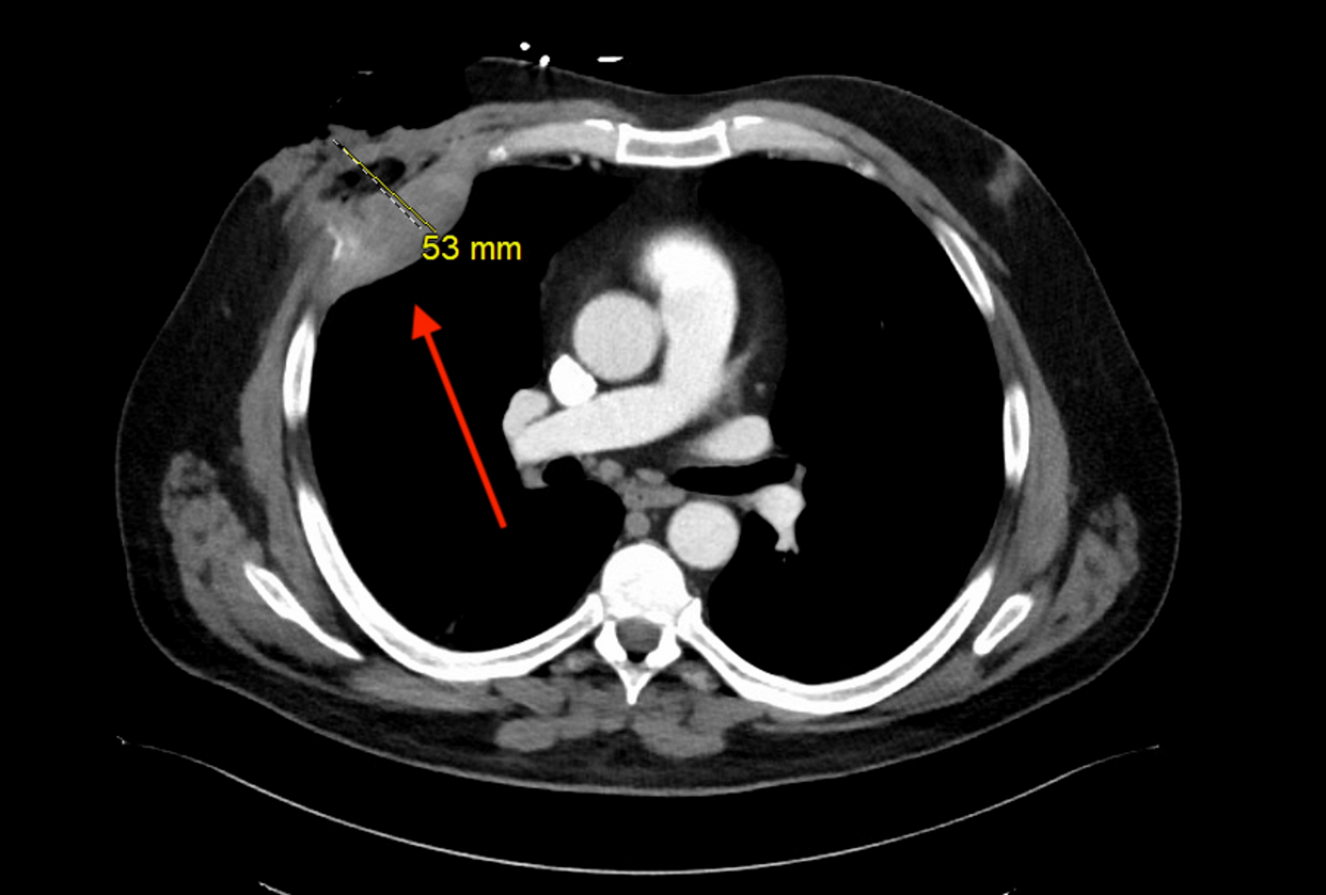
Chest CT scan with IV contrast. Contrast-enhanced axial CT image of the chest demonstrating a heterogeneous soft tissue mass involving the right anterior chest wall, with extension through the intercostal space (red arrow), measuring approximately 53 mm in maximal dimension.

**Figure 3:**
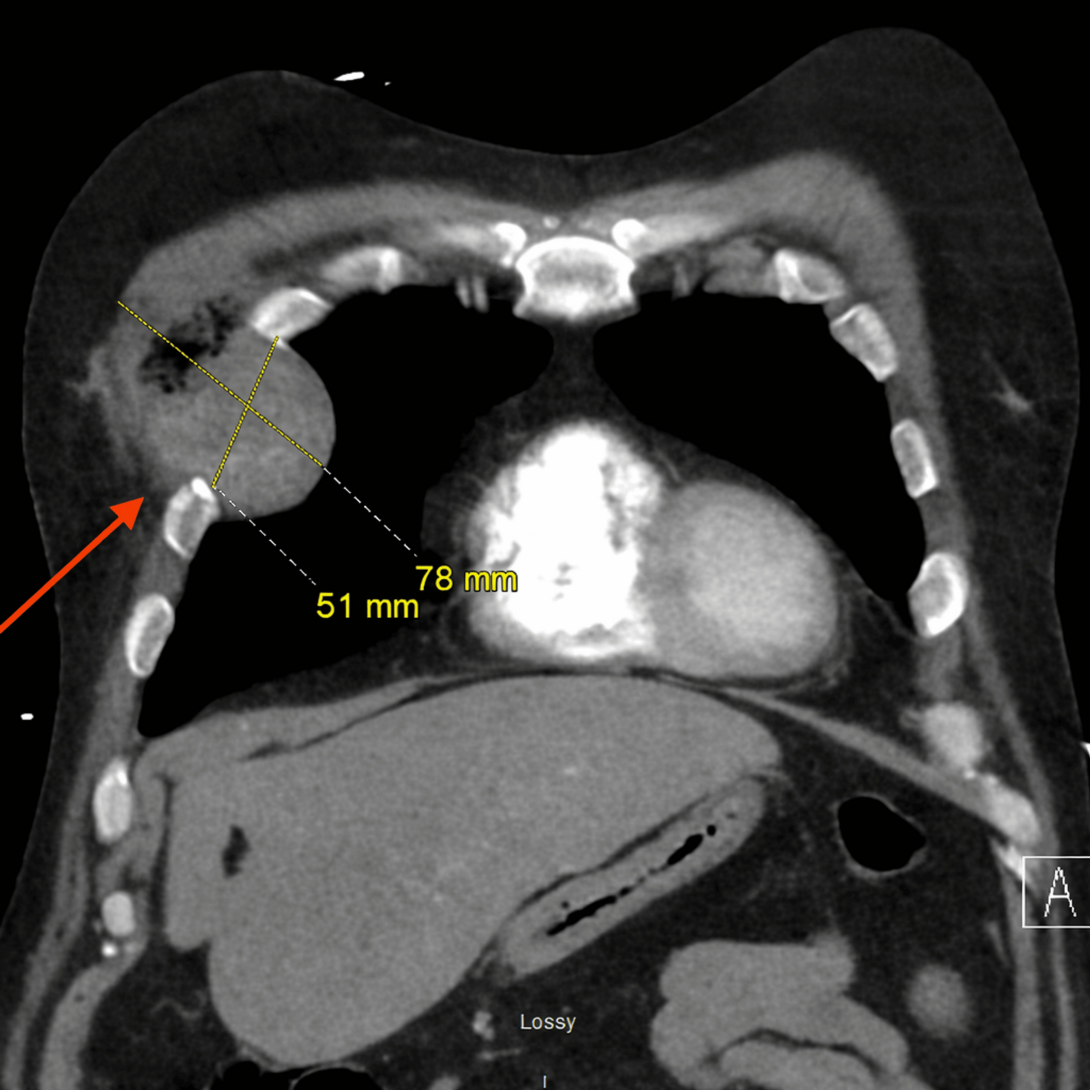
Chest CT scan with IV contrast. Coronal CT reconstruction demonstrating a lobulated soft tissue mass extending from the thoracic cavity through the right anterior chest wall into the retroareolar/subareolar region (red arrow), measuring approximately 51 × 78 mm in greatest dimensions.

Chest radiography demonstrated abnormal opacities in the right upper thoracic region (Figure 1). Subsequent contrast-enhanced CT of the chest revealed a heterogeneous soft tissue mass involving the right anterior chest wall, with extension through the intercostal space and intrathoracic involvement. On axial imaging, the lesion measured approximately 53 mm in maximal dimension (Figure 2). On coronal reconstruction, it measured approximately 51 mm × 78 mm in maximal dimensions (Figure 3). These radiologic findings correlated with the clinically palpable approximately 5 cm retroareolar/chest wall mass. The imaging appearance strongly suggested a thoracic primary lesion with direct extension into the breast region rather than an isolated breast neoplasm.

Whole-body staging evaluation was performed with contrast-enhanced CT of the chest, abdomen, and pelvis, which did not demonstrate a separate hepatic mass or other obvious infradiaphragmatic primary tumor. Brain imaging showed no intracranial metastases. PET-CT was planned as part of definitive outpatient oncologic staging following tissue diagnosis and postoperative recovery. On the basis of the available imaging, the tumor was treated clinically as at least locally advanced chest wall-invasive NSCLC, without evidence of overt brain or liver metastasis at initial staging.

No separate fine-needle aspiration cytology or dedicated preoperative percutaneous biopsy of the presumed breast lesion was performed before operative exploration. This decision was made because imaging demonstrated an invasive chest wall process with clear intrathoracic continuity rather than an isolated superficial breast lesion, and the patient had progressive pain requiring symptom-directed intervention. In our institutional practice, when imaging shows a symptomatic chest wall mass with intrathoracic extension and a surgically accessible component, operative biopsy may provide more definitive tissue architecture, broader immunohistochemical assessment, and immediate debulking for symptom relief while avoiding the risk of a limited or nonrepresentative percutaneous specimen.

Given the concerning imaging findings and progressive symptoms, the patient underwent operative exploration with a planned excisional biopsy. Intraoperatively, the tumor was identified extending through the chest wall between the sixth and seventh ribs and located deep to the pectoralis major muscle and pectoral fat, with contiguous growth into the subareolar region. Due to extensive invasion into surrounding structures, complete surgical resection was not feasible. Debulking of the accessible tumor component was performed, and tissue samples were obtained for histopathologic analysis.

Frozen section analysis demonstrated a malignant neoplasm suspicious for squamous cell carcinoma. Final histopathologic evaluation, however, demonstrated a high-grade NSCLC with immunophenotypic features of hepatoid differentiation [7]. Immunohistochemical staining showed strong diffuse positivity for HepPar-1, with focal staining for glypican-3 and arginase. The Ki-67 proliferation index was markedly elevated at approximately 80%. The diagnosis of thoracic carcinoma was supported by the morphologic pattern, the dominant intrathoracic/chest wall mass on imaging, and the absence of a liver mass or other evidence suggesting metastatic hepatocellular carcinoma. Taken together, the clinical, radiographic, and pathologic findings supported primary lung carcinoma with aberrant hepatocytic marker expression rather than metastatic hepatocellular carcinoma.

**Figure 4:**
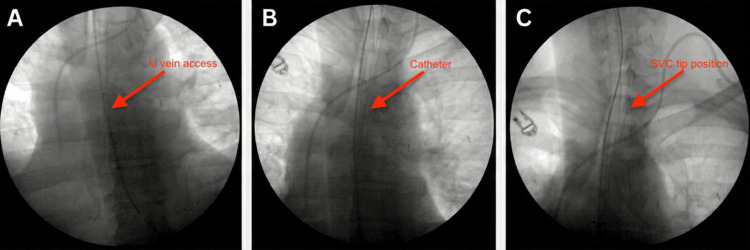
X-ray fluoroscopy. Intraoperative fluoroscopic images obtained during placement of a permanent venous access port. The left panel demonstrates internal jugular (IJ) vein access (red arrow). The middle panel demonstrates the catheter coursing through the central venous system (red arrow). The right panel demonstrates the catheter tip positioned in the superior vena cava (SVC), confirming appropriate placement for systemic therapy (red arrow).

Following diagnosis, the surgical incision was closed, and a permanent venous access port was placed via the left internal jugular vein using fluoroscopic guidance to facilitate systemic therapy (Figure 4). Given the extent of local invasion, the malignancy was considered unresectable. The patient was referred for multidisciplinary oncologic management, with planned systemic treatment consisting of platinum-based chemotherapy combined with immunotherapy, pending final comprehensive staging and molecular review.

## Discussion

Lung cancer remains the leading cause of cancer-related mortality worldwide, with NSCLC accounting for approximately 85% of cases [6]. Although cough, dyspnea, hemoptysis, and weight loss are common presenting symptoms, atypical manifestations may occur when tumors directly invade adjacent thoracic structures or present through unusual locoregional extension patterns [3,4].

Chest wall invasion occurs in a minority of lung cancers, generally in the range of 5%-8%, and is most often associated with locally advanced tumors that penetrate the parietal pleura and extend into the intercostal muscles, ribs, or overlying soft tissues [3,4]. Clinically, this may produce localized pain, chest wall fixation, or a palpable superficial mass. In the present case, the painful retroareolar lesion in this male patient was not a primary breast neoplasm but rather a chest wall-invasive thoracic malignancy with contiguous extension through the pectoralis major into the subareolar space.

This distinction is clinically important because the initial differential diagnosis of a male retroareolar mass often centers on gynecomastia or primary breast carcinoma. However, metastases or direct extension from extramammary tumors, although rare, should be considered when the lesion is fixed, painful, or radiologically continuous with the chest wall or thoracic cavity [1,2,5]. Breast-region involvement from lung malignancy is sufficiently uncommon that most of the published literature consists of case reports or small case series, and male retroareolar presentations are especially rare [5]. Compared with those prior reports, the current case is notable for clear radiographic continuity between the thoracic mass and the breast-region lesion, which allowed early recognition of a nonmammary origin.

The pathologic profile in this case required careful interpretation. Frozen section analysis initially raised concern for squamous cell carcinoma, whereas final histopathology demonstrated high-grade NSCLC with immunophenotypic evidence of hepatoid differentiation [7]. Expression of HepPar-1, glypican-3, and arginase can create a diagnostic pitfall because these markers are classically associated with hepatocellular differentiation and may also be seen in hepatoid adenocarcinoma [8,9]. Accordingly, the principal differential diagnosis included metastatic hepatocellular carcinoma and primary pulmonary hepatoid adenocarcinoma. These possibilities were reconciled by integrating pathology with the clinical and imaging findings. Specifically, the dominant disease burden arose from the thoracic cavity with direct chest wall extension, and staging imaging did not identify a separate hepatic primary lesion. Thus, the overall picture favored primary lung carcinoma with hepatocytic marker expression rather than metastatic hepatocellular carcinoma. In such cases, an aberrant hepatocytic immunophenotype should not override the anatomic and morphologic context.

The decision to proceed directly to operative biopsy rather than percutaneous sampling also warrants comment. In many patients, image-guided core biopsy would be a reasonable first-line approach. In this case, however, the symptomatic accessible component, the need for rapid tissue diagnosis, the desire for more extensive histologic architecture and immunohistochemical analysis, and the opportunity to achieve concurrent debulking for pain relief favored operative biopsy. This approach was consistent with institutional practice for selected painful chest wall lesions with substantial accessible tumor burden and clear intrathoracic continuity.

From a management standpoint, treatment of chest wall-invasive lung carcinoma depends on the extent of local invasion, nodal status, distant disease, resectability, and patient functional status [3,4]. In selected patients, en bloc resection with chest wall reconstruction may be feasible. In this patient, however, the tumor was unresectable because of extensive local invasion into surrounding structures and an ECOG performance status of 2; therefore, management appropriately shifted toward tissue diagnosis, symptom-directed debulking, venous access placement, and planning for systemic therapy. At the time of initial management, staging supported at least locally advanced unresectable disease without overt brain or liver metastases on available imaging, while definitive PET-CT-based staging was arranged through oncology.

This case has several limitations. As with many emergent or symptom-driven presentations, comprehensive staging was completed in phases rather than all at once, and final PET-CT-based TNM subclassification was deferred to the outpatient oncology workflow. In addition, because biopsy targeted the accessible chest wall component of a contiguous thoracic mass, the pathologic interpretation depended heavily on clinicoradiographic correlation. Nonetheless, this integrated approach reflects real-world practice in diagnostically complex thoracic tumors.

Clinically, this case supports a clear recommendation: fixed, painful male breast-region masses, especially those tethered to the chest wall or associated with thoracic symptoms or abnormal chest imaging, should prompt early cross-sectional imaging rather than isolated breast-focused evaluation. Earlier CT-based assessment may shorten diagnostic delay, better define the site of origin, and expedite definitive tissue diagnosis and oncologic referral.

## Conclusions

Chest wall-invasive lung carcinoma can present as a palpable retroareolar mass in male patients, representing a rare but important diagnostic consideration. Such atypical presentations may initially mimic primary breast pathology and lead to diagnostic delay if thoracic etiologies are not considered.

Comprehensive imaging and histopathologic evaluation are essential for establishing the correct diagnosis and guiding management. This case further emphasizes that early cross-sectional imaging should be strongly considered for fixed, painful male breast-region masses, particularly when the lesion appears contiguous with the chest wall.
